# Decreased Ocular Perfusion Pressure Associated With Reverse Ophthalmic Artery Flow on Transcranial Doppler Ultrasonography

**DOI:** 10.7759/cureus.60706

**Published:** 2024-05-20

**Authors:** Brian A Murillo, Anny M Cheng, Joby Tsai, Shailesh K Gupta

**Affiliations:** 1 Ophthalmology, New York Medical College, Valhalla, USA; 2 Ophthalmology, Broward Health, Fort Lauderdale, USA

**Keywords:** transcranial doppler ultrasonography, reverse ophthalmic artery flow, ophthalmodynamometry, ocular perfusion pressure, mean central retinal artery pressure, cerebrovascular occlusive disease

## Abstract

Innovative applications of clinical ocular diagnostic tools are emerging to help identify systemic disorders that extend beyond ocular diseases. Ophthalmodynamometry (ODM) is a screening tool that non-invasively determines mean central retinal artery pressure (MCRAP) and ocular perfusion pressure (OPP). Decreased OPP and MCRAP on Falck Medical Multifunctional Device (FMD, Falck Medical, Inc., Mystic, CT), along with reverse ophthalmic artery flow (ROAF) on transcranial Doppler ultrasonography (TCD), indicate increased collateral brain perfusion and possible stenosis of the ophthalmic artery or internal carotid artery (ICA). In this case report, we describe the case of a 78-year-old female with ROAF, reduced MCRAP, and OPP in the right eye, confirmed by carotid duplex of 50-79% right ICA stenosis. Early application of ODM and TCD allowed for prompt diagnosis and management with a vascular specialist.

## Introduction

Ophthalmodynamometry (ODM) is a non-invasive method to evaluate vascular perfusion dynamics in the central retinal artery (CRA) [1]. The CRA is the terminal branch of the ophthalmic artery (OA) that originates from the internal carotid artery (ICA). OA perfusion is directly related to ICA perfusion. Reverse flow in the ophthalmic artery (ROAF) is also strongly correlated with OA or ICA stenosis or occlusion [2]. Stenotic events in the OA contribute to collateral pathway diversion to keep the brain perfused. This collateral pathway contribution to overcoming stenotic events is not completely understood. In such events, transcranial doppler (TCD) ultrasonography has the potential to detect ROAF [2-4] while stenosis in OA or ICA decreases the mean central retinal artery pressure (MCRAP) and ocular perfusion pressure (OPP) on ODM.

Because of their direct anatomical connectivity, ODM evaluation of CRA can offer insights into OA perfusion and other connected vasculature. For example, the determination of OPP can be derived by calculating the difference between MCRAP and intraocular pressure (IOP). TCD can also be useful in uncovering vascular insufficiency and potential ischemic events [5]. Stenotic events in the OA or ICA decrease MCRAP and OPP, and TCD can identify ROAF associated with compromised MCRAP or reduced OPP. In this report, we identified a case of significant right ICA stenosis in an asymptomatic woman with ROAF, reduced MCRAP, and OPP. Institutional approval was waived as our single case report involves retrospective medical record review of one patient and the only interaction with the patient has been for treating the patient and does not meet the Common Rule definition of research (45 CFR 164.501). Although institutional approval was not required to publish the case details, we obtained written informed consent from the patient for the publication of her case.

## Case presentation

A 78-year-old asymptomatic woman with low-tension glaucoma status post-laser trabeculoplasty presented for a routine glaucoma follow-up. Her hypertension and hypercholesterolemia were well controlled with amlodipine and rosuvastatin. Family history was non-contributory, patient was non-diabetic and non-smoking. The best-corrected visual acuity was 20/40 OD and 20/30 OS. A slit-lamp examination of the anterior segment was unremarkable but a red-free examination detected a papillomacular bundle defect. Fundoscopic examination showed bilateral enlarged optic disc cupping and arteriolar narrowing.

Due to her glaucoma history and vascular risk factors, ODM with Falck Medical Multifunctional Device (FMD, Falck Medical, Inc., Mystic, CT) was performed. Brachial mean arterial pressure (MAP) measured with an automated blood pressure cuff was 94.3 mmHg bilaterally. FMD ODM directly measured IOP 14.9 mmHg OD and 10.4 mmHg OS; MCRAP of 46.6 mmHg OD (reduced) and 55.5 mmHg OS; and OPP of 31.7mmHg OD (reduced) and 45.1 mmHg OS.

**Figure 1:**
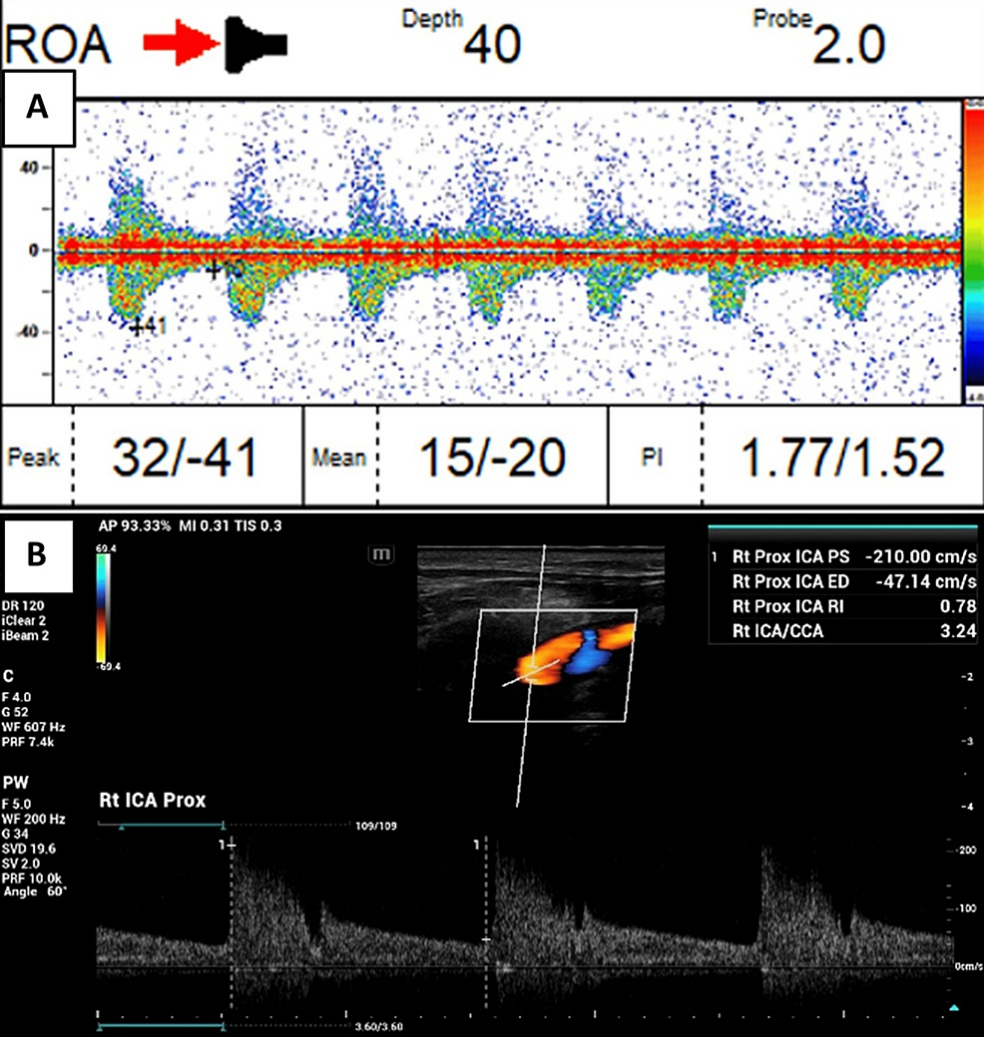
Transcranial Doppler ultrasonography (TCD) and carotid duplex scan. (A) Right ophthalmic artery (OA) waveforms by TCD below the baseline indicate blood flow away from the probe. (B) Increased right internal carotid artery (ICA) peak systolic velocity by carotid duplex indicative of right ICA stenosis.

As both MCRAP and OPP OD were reduced, further examination was conducted using TCD (Multigon, Tyler, Texas) and carotid duplex ultrasonography (Mindray, Los Angeles, California). The TCD showed a right ROAF of blood flow in the opposite direction of anterograde OA flow (Figure 1A), and the carotid duplex confirmed a significant blood flow stenosis in the right ICA. Peak systolic velocities (PSV) were 210 cm/second in the right ICA (Figure 1B), in contrast to the slower left PSV of 155.77 cm/second. Similarly, the end-diastolic velocity (EDV) was 47.14 cm/second in the right ICA, which was higher than the left EDV of 24.73 cm/second. The carotid duplex reported a 50-79% stenosis of the right ICA. Therefore, the patient was referred for further vascular intervention.

## Discussion

In the current patient, ODM suggested decreased perfusion in the vasculature between the carotid and ophthalmic arteries utilizing MCRAP, which was less than 60% of MAP. TCD further suggested an obstruction/decreased flow in the patient’s cranial vasculature due to the ROAF waveform pattern and higher pulsatility index in the right OA when compared to the left OA. In addition, our patient’s papillomacular bundle defect was likely associated with pathologic signs of glaucoma [6], which include higher parapapillary atrophy to disc ratio, lamina cribrosa degeneration, and central visual field defect. This also indicates that our patient has already had ocular ischemia due to her OA and ICA stenosis. Her ROAF signified poor perfusion to the right side of the circle of Willis, a dynamic vascular network typically maintained through collateral circulation during ICA stenosis. The reverse flow serves as a compensatory mechanism, redirecting blood flow to sustain intracranial perfusion when primary collaterals are insufficient.

This case was consistent with other studies that demonstrated the potential cerebral ischemia in ROAF patients [3]. ROAF may act as a secondary collateral pathway when primary collaterals fail to maintain proper cerebral perfusion. Hence, this reverse flow can serve as an important clinical sign of impaired cerebral hemodynamics and vasomotor reactivity [3,7]. In addition, stroke patients with ROAF have been reported to have worse outcomes than patients without ROAF [4,8]. When one segment of neurological tissue is in a compromised hemodynamic state, it redirects blood flow from ocular tissue, resulting in ROAF and a decrease in OPP [9,10]. Reduced OPP can lead to progressive vision impairment and functional decline in ocular tissues, including chorioretinal ischemia and rubeosis.

## Conclusions

Collectively, these findings render ROAF associated with compromised MCRAP or reduced OPP indicate ODM and TCD are relatively fast, inexpensive, and reproducible office-based diagnostic tests that can help determine a patient’s risk profile for systemic cerebrovascular disease. The Doppler ultrasonography confirmed the patient’s 50-79% right ICA stenosis, allowing us to intervene and prevent her from having a potential ischemic stroke and vision loss. The association between reduced MCRAP and OPP on an FMD device and ROAF on TCD are useful, non-invasive tools to screen for carotid and subsequent cerebral stenosis to prevent potential ischemic stroke and preserve vision. To conclude, both ODM and TCD can be further explored as screening tools for compromised cerebrovascular hemodynamic status.

## References

[1] Jonas JB (2003). Reproducibility of ophthalmodynamometric measurements of central retinal artery and vein collapse pressure. Br J Ophthalmol.

[2] Reynolds PS, Greenberg JP, Lien LM, Meads DC, Myers LG, Tegeler CH (2002). Ophthalmic artery flow direction on color flow duplex imaging is highly specific for severe carotid stenosis. J Neuroimaging.

[3] Tsai CL, Lee JT, Cheng CA, Liu MT, Chen CY, Hu HH, Peng GS (2013). Reversal of ophthalmic artery flow as a predictor of intracranial hemodynamic compromise: implication for prognosis of severe carotid stenosis. Eur J Neurol.

[4] Sung YF, Tsai CL, Lee JT, Chu CM, Hsu CH, Lin CC, Peng GS (2013). Reversal of ophthalmic artery flow and stroke outcomes in Asian patients with acute ischemic stroke and unilateral severe cervical carotid stenosis. PLoS One.

[5] Paulson OB (1976). Ophthalmodynamometry in internal carotid artery occlusion. Stroke.

[6] Huh MG, Shin YI, Jeong Y, Kim YK, Jeoung JW, Park KH (2023). Papillomacular bundle defect (PMBD) in glaucoma patients with high myopia: frequency and risk factors. Sci Rep.

[7] Hu HH, Sheng WY, Yen MY, Lai ST, Teng MM (1993). Color Doppler imaging of orbital arteries for detection of carotid occlusive disease. Stroke.

[8] Franjić BD, Lovričević I, Brkić P, Dobrota D, Aždajić S, Hranjec J (2021). Role of Doppler ultrasound analysis of blood flow through the ophthalmic and intracranial arteries in predicting neurologic symptoms during carotid endarterectomy. J Ultrasound Med.

[9] Sun YH, Yang YH, Huang YC, Chang CH, Hwang YS (2013). Reverse flow in ophthalmic artery helps protect the cerebrum from ischemic stroke in total carotid artery occlusion. Taiwan J Ophthalmol.

[10] Cheng SF, Zarkali A, Richards T, Simister R, Chandratheva A (2019). Carotid artery stenosis, an underestimated cause of stroke recurrence in patients with ischaemic monocular visual loss. Ann R Coll Surg Engl.

